# Embedding social accountability through transformative pedagogies: a case study from a South African physiotherapy curriculum

**DOI:** 10.1186/s12909-025-08105-7

**Published:** 2025-10-24

**Authors:** Marianne Unger, Dawn Ernstzen, Sue Statham, Adnil Titus

**Affiliations:** Division of Physiotherapy, Department of Health and Rehabilitation Sciences, Faculty of Medicine and Health Sciences, Stellenbosch University, Cape Town, South Africa

**Keywords:** Transformative learning, Responsive curriculum, Social accountability, Physiotherapy education, Social justice

## Abstract

**Background:**

Health profession curricula must produce healthcare professionals who are not only clinically competent but also socially accountable. This requires graduates to understand and respond to the health systems and social contexts within which they practice, and lecturers play an important role in facilitating the attainment of these competencies. Despite growing calls for socially accountable health professional education globally, limited research exists on how these principles are understood and implemented within physiotherapy curricula, particularly in low- and middle-income contexts where health inequities are pronounced.

**Aim:**

To explore how physiotherapy lecturers understand and integrate the principles of social justice and social accountability into a South African undergraduate curriculum and to examine the pedagogical strategies employed to foster transformative learning.

**Methods:**

A qualitative descriptive case study with an interpretivist paradigm was conducted at the Division of Physiotherapy, Stellenbosch University. The study was part of a broader multi-institutional study, and a collaborator external to the Division of Physiotherapy conducted two focus groups (*n* = 10 participants) and eight individual semi-structured interviews with permanent lecturers involved in curriculum coordination and development. Mezirow’s transformative learning theory and established theories of social justice and social accountability guided a hybrid inductive and deductive thematic analysis via AtlasTi^®^ software.

**Results:**

Two key themes emerged: (1) understanding social accountability and its underpinning elements, including professional accountability, professional identity formation, interprofessional engagement, advocacy and systems awareness, and citizenship, and (2) pedagogical strategies for transformative learning, encompassing contextual and experiential learning, structured reflection, progressive scaffolding, interprofessional collaboration, and discursive pedagogy with role modelling. While participants demonstrated a sophisticated understanding of social accountability as a multidimensional construct, significant implementation challenges were identified, including inconsistent student exposure, logistical barriers to authentic learning experiences, limited scaffolding for transformative action, and insufficient faculty development support.

**Conclusion:**

Social accountability is a recognised but complex goal within the physiotherapy curriculum. While transformative pedagogies are employed, critical gaps remain between curricular intent and implementation, particularly in scaffolding students’ progression from critical reflection to transformative action. Strengthening pedagogical coherence, enhancing faculty development, and expanding authentic experiential learning opportunities may support the development of socially responsive physiotherapy graduates equipped to address health system challenges and promote health equity.

**Trial registration:**

Not applicable.

## Introduction

The call for socially accountable health professionals is increasingly urgent globally and in South Africa (SA), where persistent health inequities and a quadruple burden of disease challenge the healthcare system [1–3]. Higher education institutions (HEIs) are expected to contribute meaningfully to social justice by producing graduates who are not only clinically competent but also critically aware of and responsive to the health systems and contexts in which they serve [4, 5].

Social accountability in health profession education can be seen as a shared commitment between universities, communities, and health professionals to address structural determinants of health, promote equity, and advocate for marginalised populations [6, 7]. As Blignaut (2021) argues, education can lay the foundations for a more just society, guided by human dignity and equity [8]. However, while competencies related to leadership, ethics, and advocacy are often embedded in physiotherapy curricula, how these are translated into pedagogical practice remains inconsistently applied across undergraduate training in SA. While systematic reviews have demonstrated that socially accountable health professional education can positively impact student learning attitudes and increase the likelihood of graduates serving disadvantaged communities [9], significant gaps remain in understanding how these principles are operationalised within specific disciplinary contexts.

Social justice, as conceptualised within health profession education, encompasses the actions required to ensure equality, fairness, and the maintenance of human rights across diverse populations [7, 10]. This concept is intrinsically linked to social responsiveness - the capacity of health professionals to recognise, understand, and act upon the social determinants of health and systemic inequities that affect patient outcomes. Social responsiveness moves beyond individual clinical competence to encompass advocacy, community engagement, and systems-level thinking that addresses root causes of health disparities rather than merely treating their consequences [11]. In the South African context, where the legacy of apartheid continues to manifest in profound health inequities, social justice and responsiveness become particularly critical competencies for health professionals who must navigate complex intersections of race, class, geography, and access to care [2].

Further challenges stem from structural and systemic constraints within the healthcare system. The public sector is overburdened by the consequences of trauma, infectious and noncommunicable diseases, and limited resources to address the rehabilitation needs of the population [12]. Moreover, calls for decolonising health curricula demand renewed attention to contextually relevant, socially just, and community-responsive education [13, 14].

Transformative learning, as theorised by Mezirow [15], offers a valuable framework for cultivating the critical consciousness required to navigate these complex realities. By engaging students in self-reflection, contextual learning, and ethical reasoning, transformative pedagogies can support the formation of socially accountable professional identities [16, 17]. A recent scoping review confirmed that transformative learning theory provides health profession educators with a valuable theoretical lens for understanding student learning in unfamiliar settings, particularly where students are encouraged to be active participants in providing care [18].

Within this context, the undergraduate physiotherapy programme at Stellenbosch University (SU) aims to support South African higher education’s transformation and social accountability agenda. The programme combines foundational knowledge with clinical and contextual learning opportunities. Despite these intentions, questions remain about how effectively the curriculum fosters students’ development as change agents equipped to address the health and rehabilitation needs of diverse communities and to advance social justice.

This study thus explores how physiotherapy lecturers at SUs understand and enact the principles of social justice and social accountability in their teaching. Specifically, it aims to do the following:

1. Explore lecturers’ perspectives on social justice, social accountability and equity and how these are embedded in the curriculum; and.
2. Explore lecturers’ perspectives on the pedagogies they use to support transformative learning and the development of socially responsive graduates.

## Methods

### Study Design and Setting

This study formed part of a larger multi-institutional study on responsive curricula in health profession education in South Africa and was conducted in the Division of Physiotherapy, Faculty of Medicine and Health Sciences at SU. This study used a qualitative descriptive case study design within an interpretivist paradigm [19, 20]. The physiotherapy division offers a four-year undergraduate degree, with a strong emphasis on foundational sciences in the first two years and clinical integration and contextual learning in the latter two years. At the time of the study, the division was engaged in a curriculum renewal process aligned with national transformation goals.

### Sampling and Participants

A homogenous purposive sampling approach [21] was used to select participants. The division has 13 permanent academic staff members, most of whom coordinate at least one module within the program. Ten staff members who were actively involved in teaching and curriculum development and provided written informed consent to participate were enrolled in the study. This group was deemed well-positioned to provide insight into how social accountability is conceptualised and implemented in the programme, as they were involved in the delivery of the curriculum content as well as the administration of the curriculum.

### Data collection

Data were collected by the umbrella project Principal Investigator, who was external to the Division of Physiotherapy, in two phases: two focus group discussions (FGDs) followed by eight individual semi-structured interviews. The FGDs aimed to explore shared understandings of social justice and social accountability, whereas interviews examined individual pedagogical approaches. The broader responsive curriculum project team developed the interview guides. All sessions were recorded and transcribed, with member checking conducted through transcript review and a post-analysis feedback session.

### Data analysis

Thematic analysis followed a hybrid approach that combined inductive and deductive coding strategies [22]. The deductive analysis [23] was guided by a conceptual framework based on Mezirow’s transformative learning theory and existing theories of social justice and accountability [24, 25].

The coding process involved several iterative steps. Initially, two authors (DE and SS) independently coded all transcripts using AtlasTi^®^ software (Scientific Software Development, GmbH, Berlin, Germany, version 9). DE conducted inductive analysis, developing an emergent codebook from the data, while SS applied the deductive theoretical framework. Initial coding achieved substantial agreement, with disagreements resolved through consensus discussion involving the whole research team. The integrated coding framework was then applied systematically across all transcripts. Codes were grouped into categories and refined through constant comparative analysis. Potential themes were identified and reviewed against the coded data and original transcripts to ensure coherence and adequate support. Theme names and definitions were refined through iterative team discussions until consensus was reached.

### Reflexivity and researcher positionality

While an external researcher conducted data collection to mitigate power dynamics, the research team acknowledges our insider position as permanent faculty members within the division under study. Although we were not directly involved in data collection, our role as program stakeholders provided valuable insights into curriculum implementation and institutional context while also introducing potential analytical biases. Our shared commitment to social accountability principles and institutional culture may have influenced how we interpreted participants’ responses and identified themes.

To address these concerns, data analysis was conducted independently by two team members using different analytical approaches, and the results were then integrated through a consensus discussion. External project team members reviewed themes and analysis to enhance analytical rigour and minimise institutional bias. We acknowledge that our perspectives as program stakeholders likely influenced our understanding of social accountability concepts and pedagogical practices. This insider knowledge enabled a deeper contextual understanding of curriculum implementation challenges and institutional dynamics but may have limited our ability to examine the taken-for-granted assumptions within our divisional culture critically. Future research would benefit from incorporating student perspectives and community stakeholder voices to triangulate faculty perceptions with those of other key stakeholders.

### Findings and Discussion

This section presents and interprets the findings from two focus group discussions in which ten physiotherapy lecturers at Stellenbosch University participated, and of those, eight participated in individual interviews. The focus groups involved six and four participants (FG1 and FG2), respectively, and all eight lecturers participated in the individual interviews (IN1-IN8).

Thematic analysis revealed two central themes: (i) understanding of social accountability and its elements, and (ii) pedagogical strategies that support transformative learning. The first theme encompasses lecturers’ conceptualisations of professional accountability, ethics of care, equity, and civic responsibility, while the second explores pedagogical approaches, including contextual learning, reflection, scaffolding, interprofessional collaboration, role modelling, and professional identity development. Each theme is presented through illustrative quotes integrated with theoretical interpretation and relevant literature to provide a critical synthesis of lecturers’ perspectives and curriculum practices. Implementation challenges are integrated within each sub-theme to highlight the gaps between curricular aspirations and practical realities.

### Understanding Social Accountability in the Curriculum

This theme explores how physiotherapy lecturers conceptualise social accountability and its foundational elements. The participants framed it as a multilayered construct encompassing professional responsibility, ethics, civic engagement, awareness of systemic inequities, interprofessional collaboration, and identity formation. These dimensions reflect the values the curriculum aspires to instil in graduates who can act as change agents within South Africa’s complex healthcare landscape.

#### Professional accountability

Lecturers described social accountability as anchored in a broader understanding of professional responsibility, which extended beyond the physiotherapy profession itself. As one participant explained: “*Professional accountability is a huge domain… it includes key stakeholders such as professional organisations*,* employers*,* the regulatory framework*,* the communities and individual patients or clients we serve*.” [FG2]. This expansive view emphasised that accountability must be *“actually directed towards the needs of that community or that person or that family”* rather than merely fulfilling professional obligations [FG1].

Participants also recognised professional accountability as an ongoing developmental process. The curriculum aims to ensure that graduates understand they have *“accountability towards yourself*,* towards the profession*,* towards your clients*,* even towards the organisation to carry on and develop”* beyond graduation [FG2]. This reflects a professional philosophy where graduates *“take the philosophy of the profession at heart*,* which means not closing a blind eye to what you see*,* but making a concerted effort”* to maintain responsibility and accountability toward their communities [FG1].

These perspectives align with Boelen and Woollard’s (2011) framework, which conceptualises accountability as encompassing the ethics of care, service orientation, and responsiveness to population health needs, and reflects a growing consensus in health profession education that clinical competence alone is insufficient for impactful practice [26].

However, participants noted difficulties in maintaining consistent messaging about professional responsibilities across different clinical sites, with “*some placements offering limited exposure to diverse professional role models*’ [IN5].

#### Professional identity formation and graduate attributes

The formation of a strong professional identity was seen as foundational for accountability and resilience in complex healthcare environments. Participants emphasised that students must first develop self-understanding before they can engage meaningfully with broader social responsibilities, as one lecturer noted: *“They need to understand themselves and their role*,* and then how they interlink with others… It’s a progressive thing”* [FG2]. This developmental progression was viewed as essential for cultivating change agents, with participants recognising that *“their ability to be a change agent needs to grow out of being very solidly based in your discipline and then having the confidence to act”* [IN6].

This perspective aligns with the literature emphasising professional identity development as a dynamic and transformative process shaped by sustained engagement in authentic contexts, critical self-reflection, and exposure to meaningful role modelling [27]. The sequential approach described by participants - from self-understanding to disciplinary grounding to confident action - reflects how students gradually internalise professional values, clarify their roles, and build the resilience needed to act ethically and responsively within complex healthcare systems.

Despite the clear development journey described, some participants noted that students in the foundational year struggled to engage meaningfully with complex professional identity concepts without sufficient clinical exposure. As one lecturer explained: *“So we now know that it’s difficult for the students to actually interpret what we teach them earlier*,* because they don’t have that context. So we want them to go out into the community*,* into the clinics*,* into the hospitals*,* into patient settings… so that they can get earlier and protective exposure to what is the end product*,* where are they going to work”* [IN8]. This highlights the need for earlier contextual experiences to solidify the sequenced approach to identity formation.

#### Interprofessional engagement

Lecturers viewed interprofessional education (IPE) as a vital mechanism for promoting collaborative practice and broader accountability to the health system. Rather than simply exposing students to other professional roles, participants emphasised IPE’s role in fostering patient-centred collaboration where professionals *“actually sit together and they do a plan for a patient… then they all work together towards a common goal for the patient”* [IN7]. This collaborative approach was seen as fundamental to shifting students away from professional silos toward shared accountability, with one participant noting that effective interprofessional practice means recognising that *“it’s not about being loyal to my profession… It’s about the patient. In addition*,* we all have a role in that activity”* [IN7]. However, logistical barriers significantly limited IPE implementation: “*The main barrier is timetables*,* different programmes*,* and also*,* that it is actually not happening in practice*” [IN8].

This patient-centred framing of IPE reflects participants’ understanding that social accountability extends beyond individual professional competence to encompass collective responsibility for health outcomes. Through structured interprofessional learning experiences, students develop the teamwork, communication, and shared problem-solving skills essential for effective rehabilitation in complex healthcare contexts where multiple professionals must coordinate care [28, 29]. Therefore, it becomes imperative to plan curricula to allow for IPE.

#### Advocacy and systems awareness

Participants emphasised the development of students’ advocacy capabilities and systems thinking as core elements of social accountability. They recognised that effective advocacy requires both understanding structural determinants of health and developing practical skills to address systemic inequities. As one participant explained, *“advocacy and leadership are part of the graduate attributes. But my feeling is that we can do more to help the students to pick up the problems that aren’t being picked up necessarily by the Department of Health”* [FG1]. This systems-level perspective was framed around developing graduates as *“change agents… someone that has some inherent responsibility for the people and communities out there… and what can I do to change the system that is maybe broken”* through systematic *“reflection*,* enquiry*,* engagement with that community”* [IN5].

However, participants identified a critical gap between raising awareness of systemic issues and teaching students how to respond effectively. While acknowledging the curriculum’s strength in exposing inequities, lecturers struggled with the practical and ethical complexities of advocacy training. The challenge was particularly acute given students’ vulnerable position in clinical hierarchies, as one participant explained: *“My ethical problem is that although they can tell me*,* they don’t have to act on it*,* because of the power differential between them and the clinicians… they’re not actually in a position to tackle the person who’s in charge”* [FG2]. This tension extended to participants uncertainty about appropriate advocacy boundaries: *“Do I teach them to be whistleblowers when I actually know they are putting themselves at a disadvantage? I can’t teach them”* [IN1]. The practical skill gap was evident in lecturers’ recognition that *“we don’t teach them that next step of what you do. Who do you rally? Who do you talk to? What are the steps to follow to make change?”* [IN3].

This finding reinforces that social accountability in physiotherapy education must extend beyond clinical training to include systems thinking and population-level care orientation [6, 27], while highlighting the need for structured approaches to advocacy skill development that recognise both students’ moral agency and professional vulnerability [25].

#### Citizenship and civic responsibility

Participants framed the purpose of the curriculum as extending beyond clinical expertise to cultivate civic-minded graduates who recognise their social responsibility. This perspective was captured succinctly by one participant who emphasised that *“this is about being a good citizen. It’s not just about being a good physio”* [IN2]. This civic orientation was seen as requiring graduates to look beyond their immediate professional sphere and recognise that *“there’s more to life than just within a 10 km radius of yourself*,* and there’s a lot of people suffering out there and that can benefit from your expertise”* [FG1]. Participants emphasised that this civic responsibility requires active engagement rather than passive awareness, noting that graduates cannot simply assume that *“somebody else will do something about it”* but must be prepared to act [FG1].

This framing is consistent with Velardo’s (2018) view that social accountability requires a shift from individual competence to collective responsibility [30]. In South Africa’s context of entrenched health inequities, this civic dimension of physiotherapy education becomes particularly critical, requiring the curriculum to instil not just clinical skills but also a sense of justice and active accountability to marginalised communities.

### Pedagogies Supporting Transformative Learning

The analysis revealed that participants’ pedagogical strategies aligned closely with Mezirow’s transformative learning phases (Table 1), suggesting an intuitive application of transformative learning principles [15] even when not explicitly referenced. The participants identified a range of approaches - both formal and informal - that aim to move students from a theoretical understanding to critical reflection and real-world action. These strategies include experiential and contextual learning, structured reflection, interprofessional collaboration, scaffolding, discussion-based teaching, and role modelling. Together, these learning activities support the development of critical consciousness, professional identity, and readiness to engage with complex health and social issues.

**Table 1:** Mapping pedagogical strategies to mezirow’s transformative learning phases

Transformative Learning Phase	Pedagogical Strategy	Implementation in Curriculum	Supporting Quote
Disorienting Dilemma	Contextual/Experiential Learning	Home visits, diverse clinical placements	“They actually do home visits… and see how they might need to adapt their treatment” [IN6]
Critical Assessment of Assumptions	Structured Reflection (DEAL model)	Post-experience reflection assignments	“We incorporate the DEAL model… describe, evaluate, and articulate learning” [IN3]
Recognition of Shared Experience	Discursive Pedagogy	Facilitated debriefing discussions	“Reflective discussion is quite often helpful, because… they need that other perspective” [FG1]
Exploration of New Roles	Interprofessional Collaboration	Joint patient care planning	“They actually sit together, and they do a plan for a patient” [IN7]
Building Competence	Progressive Scaffolding	Year-level complexity progression	“We start low and build up… with horizontal and vertical coherence” [FG2]
Integration into Life	Professional Identity Formation	Role modelling and mentorship	“Their ability to be a change agent needs to grow out of being very solidly based in your discipline” [IN6]

#### Contextual/experiential learning

Participants identified contextual and experiential learning as the most prominent and impactful pedagogical approach for fostering transformative engagement. These approaches bridge classroom knowledge with real-world complexity, enabling students to witness firsthand how theoretical concepts must adapt to contextual realities. As one lecturer described, students *“actually do home visits… and see how they might need to adapt their treatment to fit the home environment”* [IN6]. This direct exposure often challenges preconceptions, as another participant noted: *“I didn’t think a patient could get up the day after that type of surgery… then they see it and realise the patient can go home tomorrow*,* because they’re far more capable than expected”* [FG1].

Participants described increasing difficulties in securing access to diverse clinical and community-based learning environments due to lengthy approval processes and concerns about unequal student exposure across placement sites. As one lecturer explained: *“I have to then apply and give dates well in advance*,* almost sometimes a year in advance… then go to that facility and get permission there as well”* [IN5]. This creates additional concerns about equity, with participants noting: *“One of the fears is lack of equal exposure*,* and equal opportunity”* [FG2].

Such situated learning fosters embodied understanding and enhances the integration of theoretical knowledge with real-world complexity [16], creating the disorienting dilemmas that Mezirow identifies as catalysts for transformative learning. Strengthening partnerships with communities and healthcare facilities may offer pathways to overcome current barriers and ensure more consistent access to authentic experiential learning opportunities.

#### Structured reflection and feedback

Lecturers highlighted reflection as a vital pedagogical tool for transformative learning, enabling students to develop critical self-awareness and examine their professional values and assumptions. Participants emphasised the value of structured approaches, particularly the DEAL model, which helps students *“describe*,* evaluate*,* and articulate learning academically*,* civically*,* and personally”* [IN3]. The facilitated reflection process was seen as essential because students often need alternative perspectives to process complex experiences: *“that reflective discussion is quite often helpful*,* because remember*,* the student comes with a story from their perspective. So they need that other perspective sometimes to understand that perhaps the person said this*,* but could they have meant that”* [FG1].

However, participants acknowledged significant variability in how reflection was facilitated and noted that both students and staff struggled with achieving sufficient depth in reflective practice. Participants reported that reflections were often superficial and lacked guidance toward future behaviour change, with insufficient scaffolding provided by staff to support deeper processing. As one participant explained: *“The reality is that the reflection is often very limited… it’s not towards future behaviour and the becoming and the steps that follow”* [IN1]. This limitation was recognised institutionally, with participants acknowledging: *“To be fair*,* we’ve all recognised that that’s where we haven’t gone far enough. We share a lot of discussion or points or real-life situations*,* and the students are expected to reflect and they do tasks… [but it] stops with the reflection. There’s no what now?”* [FG2].

These challenges highlight that feedback literacy among staff and the integration of structured reflection require further development as intentional teaching and learning opportunities within the curriculum [32].

#### Progressive scaffolding

Participants described scaffolding as a deliberate curriculum design feature that supports the development of critical thinking, ethical reasoning, and professional accountability over time. This approach ensures *“horizontal and vertical coherence”* as students *“start low and build up”* through increasingly complex challenges [FG2]. The progression was illustrated by one participant who explained that *“on second year level*,* it would be case examples*,* and problem analysis… we would start with the impairments… Probably in the third year it is more complex… there would be aspects in there about ethical issues surrounding profession… interdisciplinary work*,* how to engage with the team”* [IN8].

However, participants noted misalignment between theoretical inputs and students’ readiness, particularly when foundational-year students encountered complex cases without sufficient contextual exposure. As one lecturer explained: *“students say they read… the case*,* but actually*,* because they didn’t have that experience*,* that exposure to the practical part of it*,* or the contextual part of it*,* they don’t really understand the case”* [FG2]. This recognition led to calls for earlier authentic exposure: *“I think there is room for exposing*,* explicitly exposing to situations*,* communities or whatever*,* from first year”* [IN7].

Such curriculum coherence is essential for embedding transformative learning principles over time [32], ensuring that students receive repeated, progressively complex opportunities to engage with and internalise critical concepts across diverse learning contexts. These challenges underscore the need for more intentional sequencing of experiential learning opportunities from the program’s earliest years.

#### Interprofessional collaboration

Participants recognised interprofessional collaboration as essential preparation for team-based healthcare delivery, with students learning to understand various professional roles and the importance of collaboration in achieving holistic outcomes.

However, meaningful implementation faced significant barriers, as one lecturer explained: *“It’s difficult to integrate it fully… The main barrier is timetables and that it’s not happening in all clinical settings”* [IN8]. Beyond scheduling conflicts, participants noted that interprofessional opportunities varied significantly across clinical sites, creating inconsistent learning experiences. The complexity of coordinating multiple programs was evident: *“the medical students are there for two weeks*,* and the physios are there for six weeks*,* and the OTs are also coming for six weeks*,* but they only overlap one week. So it’s still very haphazard”* [FG2]. Additionally, rigid clinical schedules limited responsive collaboration opportunities, with participants noting the constraints of working within *“a very tight roster”* that doesn’t allow flexibility for meaningful interprofessional engagement [IN6].

This challenge reflects broader systemic issues highlighted in the literature and demonstrates the need for institutional alignment across faculties to enable meaningful collaborative learning [28]. These barriers highlight the gap between IPE aspirations and the practical realities of coordinating multiple professional programs within existing healthcare delivery structures.

#### Discursive pedagogy and role modelling

Participants emphasised the power of facilitated dialogue and professional modelling as interconnected pedagogical approaches. Discursive pedagogy creates space for shared meaning-making, particularly in response to critical incidents where students need support in processing complex emotions and ethical tensions. As one participant noted, *“when an incident arises*,* spend an hour talking about it*,* unpacking it and reflecting on it and finding solutions*,* just in a discussion format”* to help students understand *“what kind of physio that we hope them to be one day”* [FG1].

Role modelling emerged as equally powerful, with participants recognising that *“we don’t always realise how much students observe and absorb from what they see”* [FG2]. Effective role modelling involves intentional demonstration of professional behaviours followed by reflective feedback, as one lecturer explained: *“So I do believe in role modelling. So you do have the opportunity… to actually role model certain concepts… and then be able to give them that feedback afterwards in a reflective manner”* [IN4].

However, participants noted inconsistencies in whether reflective discussions were actively facilitated and highlighted insufficient access to role models who actively embodied social accountability values in practice. As one participant observed: *“But I don’t think they get exposed enough to what it is that we do as physios. There is lack of role modelling”* [FG2]. This challenge was compounded by inconsistent interpretation among participants: *“we don’t all understand it in the same way*,* we don’t all interpret it in the same way*,* we’re not all going to role model it in the same way”* [IN4].

These approaches align with UNESCO’s emphasis on ‘tipping moments’ in transformative learning, which are often catalysed by such meaningful interpersonal engagements [17]. However, realising their full potential requires more consistent facilitation of reflective discussions and greater alignment among faculty in understanding and demonstrating social accountability principles through their own professional practice.

### Strengths and Limitations

This study provides insights into the implementation of social accountability within a specific South African physiotherapy curriculum context. The case study design effectively captured the complexities of implementation, and the hybrid thematic analysis provided both theoretical depth and contextual sensitivity. Multiple data sources enhanced trustworthiness through triangulation of faculty perspectives.

However, several significant limitations constrain the scope and transferability of findings. The single-site design limits the generalizability of findings beyond similar South African contexts, and the results may not be transferable to different institutional cultures or resource settings. Most critically, this study captures only faculty perspectives on social accountability integration. The absence of student voices represents a fundamental limitation, as lecturer perceptions of pedagogical effectiveness may differ substantially from student learning experiences. Without student perspectives, we cannot assess whether intended transformative learning outcomes are achieved or how students experience the pedagogical strategies described.

Our insider position as faculty members, while providing contextual depth, may have introduced confirmation bias in our interpretation of institutional practices. Participants may have provided socially desirable responses about social accountability integration, and our shared institutional culture may have limited critical examination of taken-for-granted assumptions.

The focus on a well-resourced, established physiotherapy program may not reflect implementation challenges in newer programs or those with different resource constraints. Additionally, the study was conducted during a period of curriculum renewal, which may have heightened participants’ awareness of social accountability concepts and influenced their responses.

Future research should prioritise student and community stakeholder perspectives to assess the actual impact of these pedagogical approaches on graduate preparedness for socially accountable practice. Longitudinal studies tracking graduate practice patterns and community engagement would provide valuable insights into the long-term effectiveness of transformative pedagogy in health professional education.

### Synthesis and Implications for Physiotherapy Education

These findings demonstrate that physiotherapy lecturer participants at Stellenbosch University have a sophisticated understanding of social accountability as a multidimensional construct encompassing professional responsibility, ethical care, health equity, and civic engagement. Lecturers utilise pedagogical strategies that align with transformative learning theory - including contextual learning, structured reflection, interprofessional engagement, and progressive scaffolding - suggesting intuitive application of evidence-based approaches to professional identity development [31].

However, significant implementation challenges create gaps between curricular intent and educational reality. Unequal student experiences across clinical sites, resource constraints limiting authentic community engagement, and insufficient scaffolding between critical awareness and practical advocacy skills reflect systemic barriers that constrain transformative learning opportunities. These challenges highlight the need for better alignment between formal curriculum design, informal learning experiences, and the hidden curriculum of institutional culture [5].

### Implications for South African physiotherapy education

Within the context of South Africa’s persistent health inequities and ongoing transformation of its healthcare system, these findings suggest several priorities for curriculum development. Faculty development must focus on deepening facilitation and feedback skills to support more effective reflective practice [32]. Programs require sustainable community partnerships that offer diverse and authentic learning experiences while addressing service delivery needs. The DISCuSS model - emphasising Diversity, community engagement, and social accountability - may offer a structured framework for strengthening curriculum-community connections [33].

Most critically, curricula must scaffold the progression from critical consciousness to transformative action. This requires intentionally designed experiences that move students beyond awareness of injustice toward practical advocacy skills and system navigation competencies [34]. Such “tipping points” - through service-learning projects, interprofessional dialogue, and structured debriefings - can bridge the gap between reflection and civic engagement [11, 35].

### Broader contextual considerations

While situated within South African healthcare contexts, these implementation challenges resonate with documented experiences across diverse geographical and resource settings. The difficulties in securing authentic clinical learning experiences reflect broader trends in medical education: rural isolation limits placement diversity, urban overcrowding restricts access to hospitals, and resource constraints fragment learning opportunities. Similarly, the tension between raising awareness and teaching responsive action mirrors the struggles reported globally in health profession programs that attempt to translate social accountability principles into practice [36].

This alignment with international experiences suggests that translating social accountability from curriculum intent to educational reality represents a universal challenge in health profession education, manifesting differently across contexts but requiring systematic, sustained approaches rather than isolated interventions. Recent guidance from international medical education bodies emphasises the need for context-specific implementation strategies while maintaining core accountability principles [36].

### Future research directions

These findings contribute to frameworks for assessing social accountability implementation by suggesting indicators such as curriculum coherence, faculty modelling behaviours, and graduate preparedness for systems-level engagement. Health profession education would benefit from longitudinal studies tracking how transformative learning experiences influence graduate practice patterns and community impact over time, particularly examining how different resource environments shape the translation of social accountability from curriculum intent to graduate capability.

The critical gap identified between awareness and action warrants particular attention in future research, especially investigating pedagogical approaches that successfully bridge this divide while recognising students’ professional vulnerability and moral agency. Such research should prioritise student and community perspectives to triangulate faculty perceptions with actual learning outcomes and community impact.

## Conclusion

This study highlights the integral role that physiotherapy educators play in fostering social accountability within undergraduate education. Lecturers at Stellenbosch University conceptualise social accountability as a complex, multidimensional construct and employ pedagogies aligned with transformative learning theory to cultivate socially responsive graduates. However, significant gaps exist between curricular intent and implementation, including inconsistent student exposure, logistical barriers, and insufficient scaffolding for transformative action.

These findings extend Mezirow’s transformative learning framework by revealing how structural and systemic constraints can interrupt the progression from critical reflection to transformative action, suggesting the need for adapted models that explicitly account for contextual barriers through intermediate steps such as collective problem-solving and advocacy skill development.

For similar South African contexts, our findings suggest prioritising faculty development in facilitation and feedback skills, developing sustainable community partnerships to expand authentic learning opportunities, and creating structured pathways for students to translate critical awareness into actionable advocacy skills. However, the absence of student perspectives in this study represents a critical limitation, and future research must incorporate learner voices to assess whether these pedagogical approaches effectively foster the intended transformative outcomes.

As South Africa continues to face stark health disparities, physiotherapy education must evolve to equip future professionals with the values, competencies, and courage to lead transformative change within complex health systems.

## Data Availability

The datasets used and/or analysed during the current study are available from the corresponding author on reasonable request.

## Abbreviations

DISCuSS: Diversity, Identify, Search, Create module (with community engagement), Sustainability, Social accountability (model)
FGD: Focus group discussion
FG (#): Focus group (number)
HEI: Higher education institution
IN (#): Interview (participant number)
IPE: Interprofessional education
SU: Stellenbosch University
SA: South Africa

## References

[1] Pillay-van Wyk V, Msemburi W, Laubscher R, Dorrington RE, Groenewald P, Glass T, et al. Mortality trends and differentials in South Africa from 1997 to 2012: second National burden of disease study. Lancet Glob Health. 2016;4(9):e642–53. 27539806 10.1016/S2214-109X(16)30113-9

[2] Mayosi B, Benatar S. Health and health care in South Africa – 20 years after Mandela. N Engl J Med. 2014;371(14):1344–53. 25265493 10.1056/NEJMsr1405012

[3] Volmink J. Reconceptualising health professions education in South Africa. S Afr J Sci. 2018;114(7/8):7–8.

[4] Dall’Alba G, Barnacle R. An ontological turn for higher education. Stud High Educ. 2007;32(6):679.

[5] Frenk J, Chen L, Bhutta ZA, Cohen J, Crisp N, Evans T, Fineberg H, Garcia P, Ke Y, Kelley P, Kistnasamy B. Health professionals for a new century: transforming education to strengthen health systems in an interdependent world. The lancet. 2010;376(9756):1923–58. 10.1016/S0140-6736(10)61854-5 21112623

[6] Buchman S, Woollard R, Meili R, Goel R. Practising social accountability from theory to action. Can Fam Physician. 2016;62(1):15–8. 26796826 PMC4721832

[7] Hixon AL, Yamada S, Farmer PE, Maskarinec GG. Social justice: the heart of medical education. Soc Med. 2013. 10.71164/socialmedicine.v7i3.2013.671.

[8] Blignaut S. Transforming the curriculum for the unique challenges faced by South Africa. Curr Perspect. 2021. 10.1007/s41297-020-00104-6.

[9] Reeve C, Woolley T, Ross SJ, Mohammadi L, Halili S, Ben, Cristobal F et al. The impact of socially-accountable health professional education: A systematic review of the literature. Med Teach. 2017;39(1) 67-73. 10.1080/0142159X.2016.1231914 27797293

[10] Sharma M, Pinto AD, Kumagai AK. Teaching the social determinants of health: A path to equity or a road to nowhere? Academic medicine Lippincott Williams and Wilkins. 2018;93:25–30. 10.1097/ACM.0000000000001689 28445214

[11] Clithero-Eridon A, Albright D, Ross A. Conceptualising social accountability as an attribute of medical education. Afr J Prim Health Care Fam Med. 2020;12(1):1–8. 10.4102/phcfm.v12i1.2213 PMC7061228 32129649

[12] Carpenter B, Nyirenda M, Hanass-Hancock J. Disability, a priority area for health research in South Africa: an analysis of the burden of disease study 2017. Disabil Rehabil. 2022;44(25):7839–47. 34783620 10.1080/09638288.2021.2000047

[13] Amosun SL, Maart S, Naidoo N. Addressing change in physiotherapy education in South Africa. South Afr J Physiotherapy. 2018;74(1) 1-4. 10.4102/sajp.v74i1.431 PMC6092759 30135922

[14] Jacobs C, Van Schalkwyk S, Blitz J, Volschenk M. Advancing a social justice agenda in health professions education. Crit Stud Teach Learn. 2020;8(2):112–31.

[15] Mezirow J. Transformative learning: theory to practice. New Dir Adult Continuing Educ. 1997;1997:74.

[16] Furze J, Black L, Peck K, Jensen GM. Student perceptions of a community engagement experience: exploration of reflections on social responsibility and professional formation. Physiother Theory Pract. 2011;27(6):411–21. 20946070 10.3109/09593985.2010.516479

[17] UNESCO. Teaching and learning transformative engagement [Internet]. 2019 [cited 2022 Feb 9]. Available from: https://unesdoc.unesco.org/ark:/48223/pf0000368961/PDF/368961eng.pdf.multi

[18] Van Schalkwyk SC, Hafler J, Brewer TF, Maley MA, Margolis C, McNamee L, et al. Transformative learning as pedagogy for the health professions: a scoping review. Med Educ. 2019. 10.1111/medu.13804. 10.1111/medu.13804 30761602

[19] Crowe S, Cresswell K, Robertson A, Huby G, Avery A, Sheikh A. The case study approach. BMC Med Res Methodol. 2011;11(1):100. Available from: http://www.biomedcentral.com/1471-2288/11/100 10.1186/1471-2288-11-100 PMC3141799 21707982

[20] Yin RK. Case study research: design and methods. 5th ed. Los Angeles: SAGE; 2014.

[21] Etikan I, Musa SA, Alkassim RS. Comparison of convenience sampling and purposive sampling. Am J Theoretical Appl Stat. 2016;5(1):1–4.

[22] Fereday J, Muir-Cochrane E. Demonstrating rigor using thematic analysis: A hybrid approach of inductive and deductive coding and theme development. Int J Qual Methods. 2006;5(1) 80-92.

[23] Braun V, Clarke V. Using thematic analysis in psychology. Qual Res Psychol. 2006. 10.1191/1478088706qp063oa.

[24] Boelen C, Woollard R. Social accountability: the extra leap to excellence for educational institutions. Med Teach. 2011. 10.3109/0142159X.2011.590248. 10.3109/0142159X.2011.590248 21774646

[25] Mezirow J. Transformative learning theory. In: Contemporary Theories of Learning. Routledge; 2018 [cited 2021 Dec 15]. pp. 114–28. Available from: https://www.taylorfrancis.com/chapters/edit/10.4324/9781315147277-8/transformative-learning-theory-jack-mezirow

[26] Green-Thompson LP, McInerney P, Woollard B. The social accountability of doctors: a relationship based framework for understanding emergent community concepts of caring. BMC Health Serv Res. 2017. 10.1186/s12913-017-2239-7. 10.1186/s12913-017-2239-7 PMC5389126 28403860

[27] Jones P. Transformative learning theory: addressing new challenges in social work education. In: Li M, Zhou Y, editors. Exploring learning & teaching in higher education. Berlin, Heidelberg: Springer-; 2015. pp. 267–86.

[28] Reeves S, Fletcher S, Barr H, Birch I, Boet S, Davies N, et al. A BEME systematic review of the effects of interprofessional education: BEME guide No. 39. Med Teach. 2016;38:656–68. 10.3109/0142159X.2016.1173663 27146438

[29] Col N, Bozzuto L, Kirkegaard P, Koelewijnvan Loon M, Majeed H, Jen Ng C, et al. Interprofessional education about shared decision making for patients in primary care settings. J Interprof Care. 2011;25(6):409–15. 22011026 10.3109/13561820.2011.619071

[30] Velardo S. Social determinants of health: a pedagogical framework for advancing the citizen scholar. Educ Citizen Soc Justice. 2018;13(3):268–79.

[31] Ryan CL, Cant R, McAllister MM, Vanderburg R, Batty C. Transformative learning theory applications in health professional and nursing education: an umbrella review. Nurse Educ Today. 2022;119:105604. 36265209 10.1016/j.nedt.2022.105604

[32] Boud D, Dawson P. What feedback literate teachers do: an empirically-derived competency framework. Assess Eval High Educ. 2021. 10.1080/02602938.2021.1910928.

[33] Goez H, Lai H, Rodger J, Brett-Maclean P, Hillier T. The DISCuSS model: Creating connections between community and curriculum - A new lens for curricular development in support of social accountability. Med Teach [Internet]. 2020;42(9):1058–64. Available from: 10.1080/0142159X.2020.1779919 10.1080/0142159X.2020.1779919 32608298

[34] Jemal A. Critical consciousness. A critique and critical analysis of the literature. Urban Rev. 2017;49(4):602–26. 29657340 10.1007/s11256-017-0411-3 PMC5892452

[35] Te M, Blackstock F, Chipchase L. Fostering cultural responsiveness in physiotherapy: curricula survey of Australian and Aotearoa New Zealand physiotherapy programs. BMC Med Educ. 2019. 10.1186/s12909-019-1766-9. 10.1186/s12909-019-1766-9 PMC6717323 31470833

[36] Abdalla ME, Taha MH, Onchonga D, Nour N, Kelly D, Harney S, et al. Exploring strategies, programs, and influencing factors for integrating social accountability into undergraduate medical education: a scoping review. BMC Med Educ. 2024. 10.1186/s12909-024-06072-z. 10.1186/s12909-024-06072-z PMC11460141 39375698

